# Late *ad libitum* feeding reverses early feed restriction-induced immune deficit in broilers

**DOI:** 10.1016/j.isci.2026.114698

**Published:** 2026-01-14

**Authors:** Fang Wang, Jiaqi Feng, Zhenxin Zhu, Shanshan Nan, Wei Jing, Min Yao, Lijing Dou, Dan Wang, Xueqiang Liu, Xiaowen Sun, Cunxi Nie

**Affiliations:** 1 Bingtuan Key Laboratory for Efficient Utilization of Non-Grain Feed Resources, College of Animal Science and Technology, Shihezi University, Shihezi, Xinjiang 832000, China; 2 School of Medicine, Shihezi University, Shihezi, Xinjiang 832000, China; 3 Animal Husbandry and Fisheries Service Development Center, Shihezi, Xinjiang 832000, China; 4 Chuang Yu Poultry Breeding Co., Shihezi, Xinjiang 832000, China; 5 Center for Animal Disease Prevention and Control, Mulei country, Xinjiang 831900, China

**Keywords:** Poultry immunology, Poultry microbiology, Agronomy, Agricultural products

## Abstract

Restricted feeding mitigates metabolic diseases and offers a cost-saving potential for broiler production. This study investigated a feeding strategy to reduce costs without compromising health. Four hundred and eighty one-day-old male broilers were divided into four groups subjected to 21-day feed restriction with different daily access times (24, 20, 16, or 12 h), followed by *ad libitum* feeding for 24 h (ADF) until 70 days. Early feed restriction (EFR) significantly decreased body weight, average daily gain, feed intake, and bursa of Fabricius index (*p* < 0.05), while suppressing intestinal Wnt/β-catenin signaling pathway and enhancing inflammatory responses. Subsequent ADF reversed these effects, restoring growth performance and immune indices to control levels. Butyric acid and butyrate-producing bacteria increased significantly (*p* < 0.05), accompanied by activated Wnt/β-catenin pathway and enriched beneficial flora. In conclusion, ADF alleviates EFR-induced inflammation and promotes immune recovery via gut microbiota and Wnt/β-catenin pathway, providing a viable cost-effective feeding strategy.

## Highlights

• Early feed restriction impairs broiler growth, immunity, and gut Wnt/β-catenin pathway
• Late *ad libitum* feeding restores immune function and reactivates gut Wnt/β-catenin pathway
• Gut microbiota and butyrate mediate recovery from early feed restriction in broilers

## Graphical abstract

**Figure:**
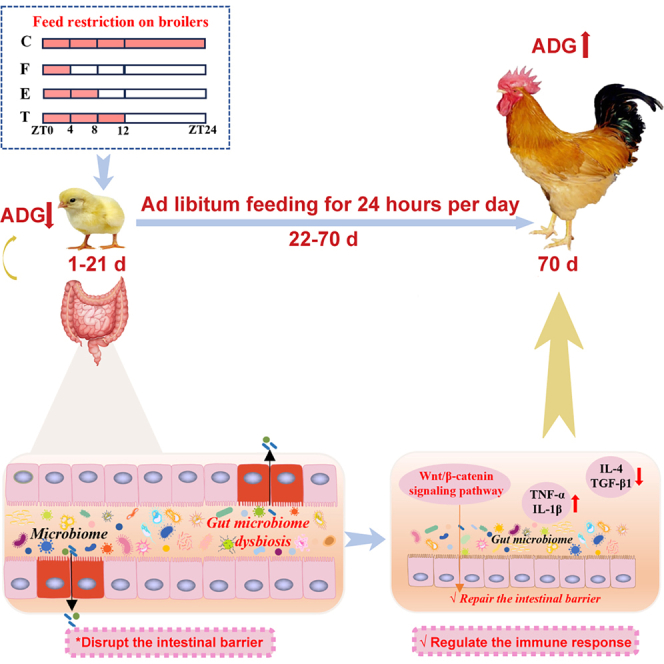

## Introduction

Changes in dietary patterns early in life are important for organ development, and early organismal development has a critical impact on growth performance and immune system maturation later in life.^1^^,^^2^ Studies have shown that feed restriction (FR) during the early stages of an animal’s life can affect growth performance, leading to compensatory growth (“catch-up growth”). In catch-up growth, animals following early FR (EFR) usually show accelerated growth, and may even grow faster than those fed normally in the early stages.^3^

Feed restriction, which mainly constitutes restrictions in quantity or time to feed access, is an effective method for lowering feed costs and increasing feed efficiency in broilers.^4^^,^^5^ Feed restriction in broilers can alter gastrointestinal tract development, influence gut microbiota structure, and improve meat quality.^6^^,^^7^ At the same time, FR can substantially reduce disease incidence and severity.^8^

The intestinal tract is important for digesting and absorbing nutrients and is one of the main immune organs in poultry.^9^ Maintaining gut health is critical in improving poultry performance.^10^ Gut microbiota is a critical factor in maintaining intestinal homeostasis. Dysbiosis of the gut flora can disrupt normal intestinal tract function, resulting in the development of intestinal inflammation, ultimately reducing broiler performance.^11^^,^^12^ Short-chain fatty acids (SCFAs), which are metabolites of the gut flora, have been shown to directly and indirectly influence immune responses.^13^ Reduced SCFAs contents in the gut are key factors in inducing intestinal inflammation in animals.^14^

The Wnt/β-catenin signaling pathway is highly conserved throughout evolution and regulates essential biological processes, such as cell proliferation, differentiation, and stem cell maintenance.^15^^,^^16^ Also, the Wnt/β-catenin signaling pathway is a master regulator of intestinal homeostasis within the intestine. It is indispensable for crypt formation and the continuous renewal of the epithelial lining.^17^ Consequently, dysregulation of this pathway is a well-established contributor to intestinal pathogenesis, leading to altered gut morphology, impaired barrier function, and a shift toward a pro-inflammatory state that can culminate in intestinal inflammation.^18^ Notably, a growing body of evidence highlights the dynamic interaction between the gut microbiota and the Wnt/β-catenin pathway.^19^^,^^20^ Specifically, microbial metabolites such as SCFAs, including butyrate, have been identified as key mediators that modulate Wnt/β-catenin signaling activity, thereby influencing intestinal health and systemic immunity.^21^^,^^22^

Feed restriction can reduce feeding costs and affect broiler health, but most studies have focused on reducing feed quality, whereas few have focused on limiting feeding time for economic benefits and intestinal development of broilers.^7^^,^^23^ It is also unclear whether EFR, followed by *ad libitum* feeding (ADF), affects broiler growth and immunity. In the current study, yellow-feathered broilers were restricted to different lengths of ADF per day to 21 days, which was followed by a period of all-day ADF until 70 days; changes in gut health and microbiota were determined. This study aimed to explore the effect of FR on the intestinal immunity of broilers and determine whether this would improve broiler health after ADF to provide new feeding methods for the production of yellow-feathered broilers.

## Results

### Growth performance

There was a significant reduction in final body weight (BW), average daily gain (ADG), average daily feed intake (ADFI), in broiler chickens subjected to restricted feeding (*p* < 0.05) in the EFR stage (Table 1). Feed conversion ratio (FCR) was significantly lower in the T group (access for feeding for 12 h per day) than in the control group (C) (*p* < 0.05). Importantly, a significant interaction (21 days × 70 days) was observed for both ADG (*p* = 0.019) and ADFI (*p* < 0.001). This finding confirms that growth patterns during the ADF phase are influenced by early feeding strategies. All growth indices were similar across the groups at the ADF stage (*p* > 0.05). This shows that the ADF stage effectively compensated for the early growth impairment caused by EFR.

**Table 1:** Effects of EFR and ADF stages on growth performance of broilers

Items	Treatment (1–21 days)				SEM (1–21 days)	Treatment (22–70 days)				SEM (22–70 days)	*p* value		
	C	F	E	T		C	F	E	T		1–21 days	22–70 days	(1–21 days × 22–70 days)
Initial BW, g	34.17	34.92	34.83	34.50	0.18	NA	NA	NA	NA	NA	0.436	NA	NA
Final BW, g	522.50^a^	500.00^a^	482.50^a^	452.50^a^	5.08	3,129.25	3,021.25	3,034.00	3,011.83	24.77	<0.001	0.441	0.211
ADG, g/d	22.82^a^	21.69^a^	21.49^a^	19.85^a^	0.24	53.25	51.45	52.05	53.23	0.52	<0.001	0.557	0.019
ADFI, g/d	42.42^a^	40.15^a^	38.81^a^	33.66^a^	0.62	106.55	109.00	108.14	106.99	0.67	<0.001	0.585	<0.001
FCR	1.86^a^	1.86	1.81	1.70^a^	0.02	2.00	2.13	2.08	2.01	0.03	0.011	0.415	0.181

a Indicates significant differences at *p* < 0.05 (*n* = 6) as determined by two-way ANOVA followed by Tukey’s HSD test. The *p* values for the main effects of feeding regimen at each time point (1–21 days, 22–70 days) and (1–21 days × 22–70 days) interaction are shown.

### Immune organ indices

On day 21, the liver index was significantly lower in group F (access for feeding for 20 h per day) and the spleen index was significantly lower in group T compared to the control group (*p* < 0.05). The bursa Fasciola index was reduced (*p* < 0.05) after EFR. In addition, a highly significant interaction (21 days × 70 days) was found for the bursa of Fabricius index (*p* = 0.001). This indicates that the recovery pattern of this key immune organ was significantly affected by EFR. Following the ADF period, no significant differences in immune organ indices were detected among the groups at 70 days (*p* > 0.05), suggesting comprehensive recovery of immune organ development (Table 2).

**Table 2:** Effects of EFR and ADF stages on immune organ indices of broilers

Items	Treatment (21 days)				SEM (21 days)	Treatment (70 days)				SEM (70 days)	*p* value		
	C	F	E	T		C	F	E	T		21 days	70 days	(21 days × 70 days)
Liver index	3.05^a^	2.87^a^	3.34	3.21	0.07	1.73	1.89	1.84	1.62	0.06	0.046	0.462	0.840
Thymus index	0.26	0.23	0.23	0.22	0.01	0.32	0.33	0.35	0.33	0.01	0.371	0.899	0.437
Spleen index	0.14^a^	0.12	0.12	0.11^a^	0.01	0.14	0.15	0.14	0.13	0.01	0.039	0.927	0.337
Bursa of Fabricius index	0.31^a^	0.27^a^	0.25^a^	0.26^a^	0.01	0.15	0.17	0.18	0.15	0.01	<0.001	0.194	0.001

a Indicates significant differences at *p* < 0.05 (*n* = 6) as determined by two-way ANOVA followed by Tukey’s HSD test. The *p* values for the main effects of feeding regimen at each time point (21 days, 70 days) and (21 days × 70 days) interaction are shown.

### Serum inflammatory cytokines

**Figure 1:**
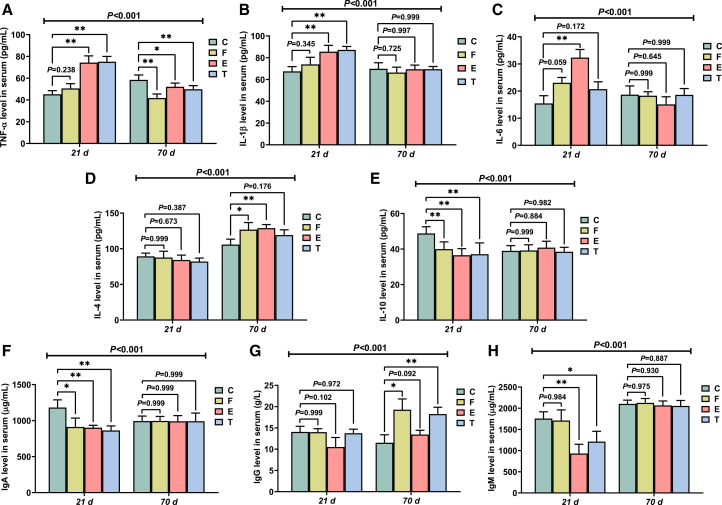
Effects of early feed restriction (EFR) and *ad libitum* feeding for 24 h (ADF) stages on serum inflammatory cytokine levels of broilers (A) TNF-α. (B) IL-1β. (C) IL-6. (D) IL-4. (E) IL-10. (F) IgA. (G) IgG. (H) IgM. Values are presented as mean ± SEM (*n* = 6). ∗ and ∗∗ indicate significant differences compared to the control group (C) at *p* < 0.05 and *p* < 0.01, respectively. The *p* value for the (21 days × 70 days) interaction from the two-way ANOVA is displayed above the respective graphs.

Significant interactions (21 days × 70 days) were observed for serum inflammatory cytokines (*p* < 0.001). On day 21, serum TNF-α and IL-1β in the groups E and T and IL-6 in the group E (access for feeding for 16 h per day) were higher than in group C (*p* < 0.01) (Figures 1A–1C). The concentration of IgA in groups F (*p* < 0.05), E, and T (*p* < 0.01) and IgM in groups E (*p* < 0.01) and T (*p* < 0.05) were lower than in group C (Figures 1F and 1H). Compared to control group, serum IL-10 was observed decreased in the EFR groups (*p* < 0.01) (Figure 1E). However, by day 70, The EFR groups showed increased serum levels of anti-inflammatory factors (IL-4 and IL-10) and immunoglobulins (IgA and IgM), while levels of pro-inflammatory cytokines (TNF-α, IL-1β, and IL-6) were similar to or lower than those in the control group (Figure 1).

### Ileum histomorphology

**Figure 2:**
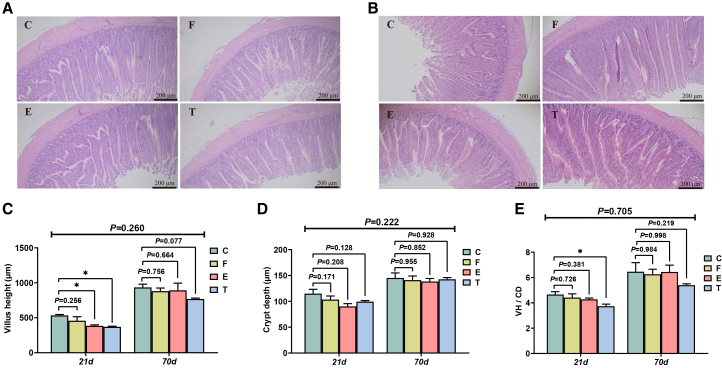
Effects of EFR and ADF stages on ileum morphology of broilers (A) H&E staining of ileum in EFR stage. (B) H&E staining of ileum in ADF stage. (C) Villus height (VH). (D) Crypt depth (CD). (E) VH/CD. Values are presented as mean ± SEM (*n* = 6). ∗ and ∗∗ indicate significant differences compared to the control group (C) at *p* < 0.05 and *p* < 0.01, respectively. The *p* value for the (21 days × 70 days) interaction from the two-way ANOVA is displayed above the respective graphs.

Ileal villus height (VH) was markedly reduced on day 21 after restricted feeding (Figures 2A and 2C). Group E had lower VH to crypt depth (VH/CD) ratio than group C (*p* < 0.05). Although the interaction effect for these morphological parameters was not statistically significant, the main effect of feeding restriction was evident during the EFR phase (*p* > 0.05). After ADF, VH, CD, and VH/CD ratio were no significantly different in all groups on day 70 (*p* > 0.05) (Figures 2B–2E).

### Gene expression and protein abundance in the ileum

**Figure 3:**
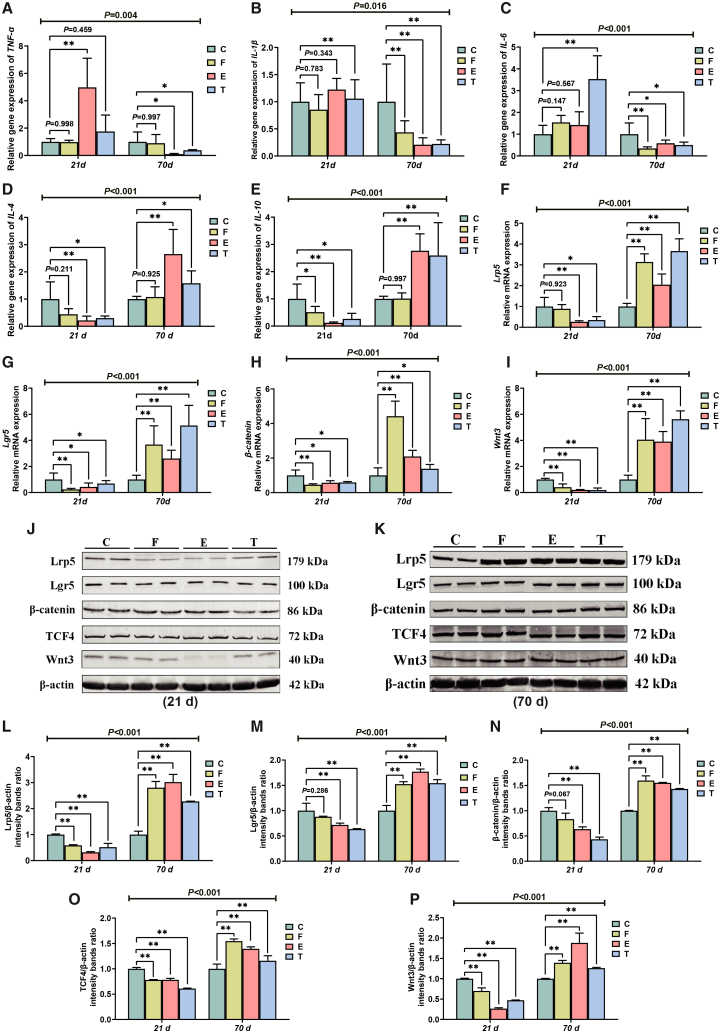
Effects of EFR and ADF stages on intestinal immune function of broilers (A–E) Ileal inflammatory cytokine gene expression. (F–I) Relative mRNA expression of *Lrp5*, *Lgr5*, *β-catenin*, and *Wnt3* in the ileum. (J–P) Relative protein levels of the Wnt/β-catenin signaling pathway. Values are presented as mean ± SEM (*n* = 6). ∗ and ∗∗ indicate significant differences compared to the control group at *p* < 0.05 and *p* < 0.01, respectively. The *p* value for the (21 days × 70 days) interaction from the two-way ANOVA is displayed above the respective graphs.

Significant interactions (21 days × 70 days) were detected in the expression levels of inflammation-related genes and Wnt/β-catenin pathway-related genes and proteins in the ileum (*p* < 0.05) (Figure 3). This shows that the pathway was dynamically regulated throughout the experiment. On day 21, EFR downregulated the gene and protein expression of key pathway components, including Wnt3, Lrp5, Lgr5, β-catenin, and TCF4. Remarkably, this suppression was completely reversed after ADF, with the treatment groups displaying a significant upregulation of these molecules at both the gene and protein levels by day 70 (Figures 3F–3P). A similar pattern was observed in the expression of inflammatory cytokine genes: proinflammatory factors (*TNF-α*, *IL-1β*, and *IL-6*) showed increased expression during the EFR stage, while anti-inflammatory factors (*IL-4* and *IL-10*) exhibited increased expression during the ADF stage (Figures 3A–3E).

### Cecal microbiota

**Figure 4:**
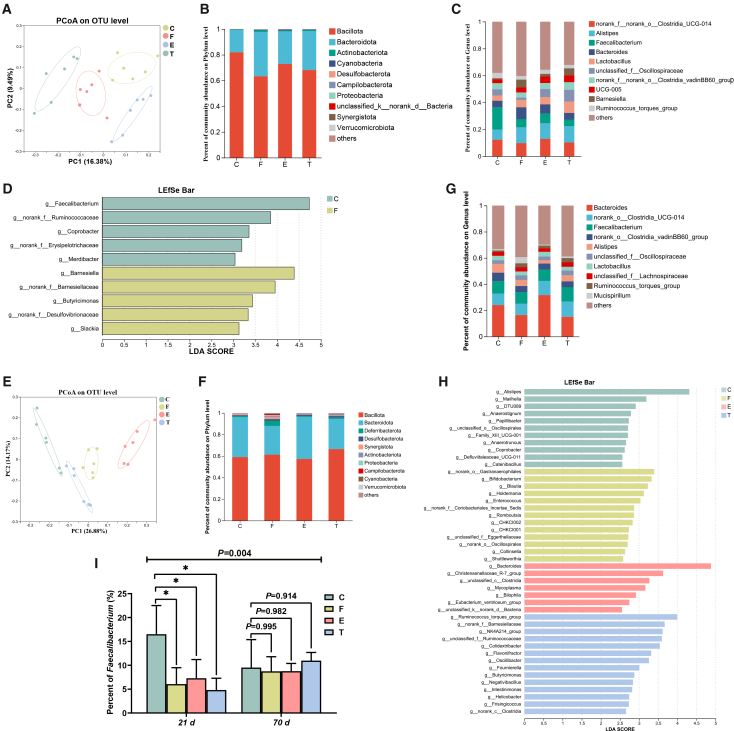
Effect of EFR and ADF stages on changes in the cecal microbiota of broilers (A) PCoA on OUT level at the EFR stage. (B) The composition of microbial communities at the phylum level at the EFR stage. (C) The composition of microbial communities at the genus level at the EFR stage. (D) LDA score for LEfSe analysis at the EFR stage. (E) PCoA on OUT level at the ADF stage. (F) The composition of microbial communities at the phylum level at the ADF stage. (G) The composition of microbial communities at the genus level at the ADF stage. (H) LDA score for LEfSe analysis at the ADF stage. (I)The percentage of *Faecalibacterium* at the EFR and ADF stages. Values are presented as mean ± SEM (*n* = 6). ∗ and ∗∗ indicate significant differences compared to the control group at *p* < 0.05 and *p* < 0.01, respectively. The *p* value for the (21 days × 70 days) interaction from the two-way ANOVA is displayed above the respective graphs.

The cecal microbial community structure was analyzed via 16S rRNA sequencing (Figure 4). PCoA results on days 21 and 70 revealed that the composition of the cecal microbiota differed among the groups (Figures 4A and 4E). Bacillota (formerly known as Firmicutes) and Bacteroidota were observed to be predominant both at the EFR and ADF stages (Figures 4B and 4F). The LEfSe analysis indicated significant differences in the abundance of 12 and 45 genera among the groups at the EFR and ADF stages, respectively (Figures 4D and 4H). Notably, a significant interaction (21 days × 70 days) was detected for the genera *Faecalibacterium* (*p* = 0.004). The relative abundance of *Faecalibacterium*, a beneficial butyrate-producing genus, was significantly reduced by EFR at day 21 but recovered to levels comparable with the control group after ADF (Figure 4I).

### Cecal SCFAs contents

The contents of acetic acid in groups E and T, propionic acid in group T, butyric acid (BA) in group F, and isobutyric acid in group F, were significantly lower than those in group C (*p* < 0.05) on day 21 (Table 3). Restricted feeding for 21 days resulted in a significant reduction in the level of isovaleric acid in the cecum of broilers (*p* < 0.05). Significant interactions (21 days × 70 days) were detected for acetic acid, propionic acid, and isovaleric acid (*p* < 0.001). After ADF, there was an increase in acetic and propionic acids in group E (*p* < 0.05) and a decrease in isovaleric acid content in group T (*p* < 0.05), compared to the control group on day 70. BA was remarkably higher in the treated groups (*p* < 0.05), indicating the restoration of the production of this beneficial fatty acid. This pattern of change corresponded well with the observed shifts in the relative abundance of *Faecalibacterium* in the cecum.

**Table 3:** Effects of EFR and ADF stages on cecal SCFAs contents of broilers

Items	Treatment (21 days)				SEM (21 days)	Treatment (70 days)				SEM (70 days)	*p* value		
	C	F	E	T		C	F	E	T		21 days	70 days	(21 days × 70 days)
Acetic acid	23.36^a^	23.46	18.62^a^	20.61^a^	0.59	22.16^a^	24.11	27.11^a^	22.05	0.67	0.002	0.007	<0.001
Propionic acid	6.57^a^	7.11	5.77	5.24^a^	0.29	7.05^a^	7.58^a^	8.34^a^	7.26	0.17	0.023	0.752	0.001
Butyric acid	6.64^a^	5.88^a^	5.94	5.74^a^	0.12	5.92^a^	6.77^a^	6.83^a^	6.77^a^	0.13	0.013	0.016	0.242
Isobutyric acid	0.28^a^	0.22^a^	0.26	0.26	0.01	0.43	0.39	0.40	0.41	0.02	0.005	0.180	0.239
Valeric acid	0.29	0.31	0.29	0.29	0.01	0.51	0.51	0.50	0.49	0.01	0.564	0.616	0.721
Isovaleric acid	0.37^a^	0.26^a^	0.30^a^	0.27^a^	0.02	0.41^a^	0.44	0.43	0.29^a^	0.02	<0.001	0.001	<0.001

a Indicates significant differences at *p* < 0.05 (*n* = 6) as determined by two-way ANOVA followed by Tukey’s HSD test. The *p* values for the main effects of feeding regimen at each time point (21 days, 70 days) and (21 days × 70 days) interaction are shown.

### Spearman correlation analysis

**Figure 5:**
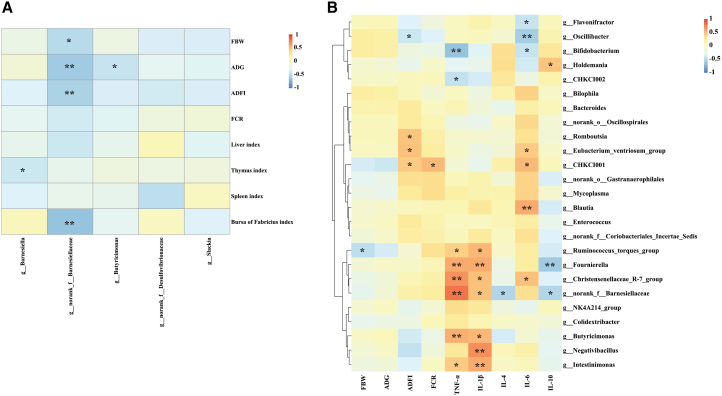
Spearman’s correlations between the gut microbiota and phenotypes (A) At the EFR stage. (B) At the ADF stage. ∗ and ∗∗ indicate significant differences at *p* < 0.05 and *p* < 0.01, respectively.

The relative abundance of *norank_f__Barnesiellaceae* was negatively correlated with FBW, ADG, ADFI, and the bursa of the Fabricius index at 21 days (Figure 5A). The relative abundance of *Barnesiella* was significantly negatively correlated with the thymus index, and the relative abundance of *Butyricimonas* was significantly negatively correlated with ADG (*p* < 0.05). Moreover, the relative abundance of *Romboutsia* was strongly positively correlated with ADFI (*p* < 0.05), the relative abundance of *CHKCI002* and *Bifidobacterium* were negatively correlated with serum TNF-α content (*p* < 0.05), the relative abundance of *Holdemania* was positively correlated with serum IL-10 content (*p* < 0.05), and the relative abundance of *Bifidobacterium*, *Oscillibacter* and *Flavonifractor* was negatively correlated with serum IL-6 content (*p* < 0.05) on day 70, suggesting a beneficial role of these microbes during the recovery phase (Figure 5B).

## Discussion

The purpose of FR in broilers is to prevent metabolic disorders and diseases during growth. However, the increasing cost of feed has focused the attention of producers on FR as a means of reducing feed costs.^24^ In the current study, we implemented a 21-day FR protocol during the early growth phase of broiler chickens to evaluate its effects on feed costs and growth performance.^5^ Specifically, we aimed to determine whether EFR would adversely affect broiler development and whether subsequent ADF would effectively reverse the negative effects during the later growth period.

The study indicated that short-term EFR markedly reduced broiler growth performance. This may be caused by EFR-induced alterations in cecal SCFAs contents, which are known to stimulate intestinal secretion of appetite-suppressing peptides and potentially reduce appetite in broilers, ultimately leading to decreased BW gain.^25^ No marked effect of EFR on growth performance was found after the ADF stage. Correspondingly, we also observed that there was no significant difference in cecal SCFAs contents among the groups on 70 days. Implementing EFR can effectively reduce feed consumption and ultimately lower feeding costs without affecting final broiler BW.^26^^,^^27^

Many studies have shown that cytokines IL-1β, IL-6, and TNF-α have pro-inflammatory effects, whereas IL-4 and IL-10 are considered to regulate anti-inflammatory effects.^28^ We found that broilers in the EFR groups had higher contents of IL-1β, IL-6, and TNF-α and lower contents of IL-4 and IL-10 than those in group C. On day 70, the content of pro-inflammatory cytokines was reduced and the content of anti-inflammatory cytokines was restored in the treated groups compared to group C. It was proved that the immune performance of broilers was impaired by EFR, but this effect could be reversed by ADF. This result was also consistent with the findings on the gene expression of *IL-1β*, *IL-6*, *TNF-α*, *IL-4*, and *IL-10* in the ileum.

Degeneration of the thymus and other immune organs affects immune function and plays a key role in animal health.^29^ The intestine, particularly the ileum, is an important immune organ in poultry.^9^ Dysfunction of the intestinal barrier has become widely identified as a major factor in the pathogenesis of numerous diseases.^30^ In the present study, the effect on intestinal histomorphology was used to evaluate intestinal barrier function in broilers during both the EFR and ADF phases.^31^ On day 21, a marked reduction in ileal VH and CD was observed in the EFR group, relative to group C. However, on day 70, VH, CD, and VH/CD ratio in groups F and E were similar to the control group.

Our results demonstrate that the severity of intestinal compromise followed a dose-response relationship with the duration of early FR. Although all restricted groups were affected, the T group (12-h access) exhibited the most severe impairment, showing the greatest suppression in growth performance, the highest levels of systemic pro-inflammatory cytokines (IL-1β, IL-6), and the most significant damage to ileal morphology (shortest VH, lowest VH/CD). This suggests a 12-h fast may approach a critical threshold that severely challenges intestinal barrier integrity and immune homeostasis.^18^ Notably, despite overall recovery after ADF, the persistently lower VH in the T group at day 70 indicates that such prolonged restriction may cause residual, incompletely reversible damage.^31^ Therefore, while a 12-h restriction minimizes feed cost, a 16 or 20-h regimen likely offers a superior balance between economic efficiency and the preservation of long-term gut health.

Intestinal microbiota has an essential effect on the regulation of animal immune function and maintaining homeostasis is fundamental to intestinal health.^32^ SCFAs, which are often considered as useful for the host, consist of metabolites produced by gut microorganisms.^13^ When the abundance of SCFAs-producing bacteria or the concentration of SCFAs decreases, dysbiosis of the intestinal microbiota occurs, resulting in the gut inflammatory response.^13^^,^^33^ Our study provides compelling evidence that the gut microbiota-Wnt/β-catenin axis is a key mechanism through which EFR impairs, and subsequent ADF restores, intestinal health in broilers. The Wnt/β-catenin pathway is a master regulator of intestinal homeostasis, governing epithelial proliferation, differentiation, and barrier integrity.^15^^,^^17^ We demonstrated that EFR significantly suppressed this pathway, as evidenced by reduced gene and protein expression of key components, such as Wnt3, Lrp5, Lgr5, and β-catenin. This suppression likely contributed to the observed ileal morphological damage and compromised barrier function. Importantly, the subsequent ADF period not only normalized growth performance but also strongly reactivated the Wnt/β-catenin pathway, suggesting its pivotal role in intestinal recovery. We propose that the gut microbiota and its metabolites, particularly BA, play an instrumental role in this process. EFR induced a state of microbial dysbiosis, characterized by a reduction in beneficial, BA-producing bacteria like *Faecalibacterium* and a decrease in cecal BA levels. Butyric acid, a key microbial metabolite, has been increasingly recognized not only for its anti-inflammatory properties but also as a potential modulator of the Wnt/β-catenin pathway.^21^^,^^22^^,^^34^ *Faecalibacterium* is an important BA-producing gut bacteria and has anti-inflammatory properties that can lower inflammation by releasing metabolites that improve intestinal barrier function.^35^ The recovery of *Faecalibacterium* abundance and the significant increase in cecal BA during ADF perfectly coincided with the reactivation of the Wnt/β-catenin signaling. This temporal correlation strongly suggests that microbiota-derived BA may serve as a signaling molecule that restores Wnt/β-catenin activity, promoting epithelial repair and immune homeostasis.^19^^,^^20^ Our findings align with recent studies indicating a functional interplay between specific gut bacteria, their metabolic output, and critical host signaling pathways in maintaining intestinal health.^21^^,^^22^^,^^36^

Gut damage is typically indicated by a reduction in the number of beneficial bacteria in the intestinal flora.^30^ In the present study, several bacterial species were highly negatively correlated with the growth performance and immune organ indices under EFR conditions. Of these, *Barnesiella* and *Butyricimonas* were observed increase in abundance in organisms with increased inflammatory responses.^37^^,^^38^ Consistently, the increased abundance of several of these genera in the EFR groups at 21 days was accompanied by a marked reduction in broiler growth performance. At the ADF stage, we further focused on the increased abundance of potentially beneficial gut microbiota in the treated groups were negatively correlated with serum pro-inflammatory factor contents and were strongly positively correlated with growth performance. *Romboutsia* and *CHKCI002* have the ability to produce BA, which is consistent with the ADF stage, where BA production was increased in group F compared to the control group.^39^^,^^40^ Anti-inflammatory properties of *Bifidobacterium*, as well as *norank_o_Gastranaerophilale*, *Holdemania*, and *Romboutsia* were observed to increase in abundance in the presence of an inhibited inflammatory response.^38^^,^^41^^,^^42^^,^^43^ *Oscillibacter* and *Flavonifractor* have been proven to play an important role in maintaining vascular health, and *Flavonifractor* can inhibit the expression of *TNF-α* in adipose tissue.^44^^,^^45^^,^^46^ These roles are consistent with the results found in the current study. The results indicate that ADF after EFR results in an increase in a variety of beneficial bacteria in the broiler gut, suggesting that FR increases the inflammatory response in the broiler gut, but this can be improved by ADF.

In conclusion, EFR initially reduced growth performance and impaired intestinal barrier function, as evidenced by increased pro-inflammatory bacteria and disruption of the Wnt/β-catenin pathway. However, subsequent ADF effectively reversed these adverse effects. A 49-day ADF intervention enhanced immune competence, increased beneficial bacteria (e.g., *Blautia* and *Bifidobacterium*), and reactivated the Wnt/β-catenin signaling pathway. This improved gut stability. Importantly, the combined feeding strategy of 21 days of EFR followed by ADF successfully reduced feed costs without negatively affecting the overall growth performance of broilers at 70 days. This aligns with the study’s goal of achieving cost-effective and sustainable broiler production.

### Limitations of the study

While this study demonstrates that late ADF can reverse early FR-induced immune deficits in broilers through modulation of the gut microbiota and Wnt/β-catenin signaling, certain limitations warrant consideration. The exclusive use of male broilers precludes evaluation of potential sex-specific responses to the feeding regimen. Furthermore, although strong associations were observed between microbial shifts, pathway activation, and immune recovery, the evidence remains largely correlative. Further targeted intervention studies are needed to elucidate causal mechanisms. The study design included only two sampling time points, which may not capture the full dynamic progression of damage and recovery. Finally, the focus on specific immune and metabolic parameters leaves open questions regarding broader physiological impacts, such as long-term health and product quality outcomes.

## Resource availability

### Lead contact

Any additional information and requests for resources and reagents should be directed to and will be fulfilled by the lead contact, Cunxi Nie (niecunxi@shzu.edu.cn).

### Materials availability

This study did not generate new unique reagents.

### Data and code availability

• The raw sequencing data generated in this study have been deposited in the Sequence Read Archive of the National Center for Biotechnology Information under the BioProject accession number PRJNA1233905.
• This study does not report original code.
• Any additional information and requests for resources and reagents used in this article should be directed to the lead contact.

## Acknowledgments

This study was supported by the 10.13039/501100001809National Natural Science Foundation of China (32360840), the Tianshan Talents Project of Xinjiang (2023TSYCCX0121), and the Science and Technology Program of XPCC (2023AB007-01).

## Author contributions

F.W.: writing – original draft, methodology, investigation, formal analysis, data curation, and conceptualization; J.F.: methodology and data curation; Z.Z.: investigation; S.N.: formal analysis, and writing – review & editing; W.J.: validation; M.Y.: supervision; L.D.: resources; D.W.: conceptualization; X.L.: investigation; X.S.: validation; C.N.: writing – review & editing, supervision, and funding acquisition.

## Declaration of interests

The authors declare that they have no known competing financial interests or personal relationships that could have appeared to influence the work reported in this study.

## STAR★Methods

### Key resources table

**Table:** 

REAGENT or RESOURCE	SOURCE	IDENTIFIER
**Antibodies**		

Rabbit polyclonal anti-Lrp5	Proteintech	Cat# 24899-1-AP; RRID: AB_2879786
Rabbit polyclonal anti-Lgr5	Proteintech	Cat# 30007-1-AP; RRID: AB_3086207
Mouse monoclonal anti-TCF4	Proteintech	Cat# 68607-1-Ig; RRID: AB_3085302
Rabbit polyclonal anti-Wnt3	Proteintech	Cat# 28156-1-AP; RRID: AB_3669649
Mouse monoclonal anti-β-catenin	Proteintech	Cat# 66379-1-Ig; RRID: AB_2857358
Mouse monoclonal anti-β-actin	Proteintech	Cat# 66009-1-Ig; RRID: AB_2687938
Anti-mouse IgG (H + L) (DyLight 680 Conjugate)	Cell Signaling Technology	Cat# 5470; RRID: AB_10697506
Anti-rabbit IgG (H + L) (DyLight 800 Conjugate)	Cell Signaling Technology	Cat# 5151; RRID: AB_10691475

**Chemicals**		

Radioimmunoprecipitation assay (RIPA)	Solarbio	Cat#: R0010
Phenylmethanesulfonyl fluoride (PMSF)	Solarbio	Cat#: P0100
Sulfate-polyacrylamide gel electrophoresis (SDS-PAGE)	Coolaber	Cat#: SK60181
Polyvinylidene fluoride (PVDF)	Servicebio	Cat#: G6047-50-0.45

**Critical commercial assays**		

Chicken TNF-α ELISA Kit	Shanghai Enzyme-linked Biotechnology Co., Ltd	Cat#: ml002790
Chicken IL-1β ELISA Kit	Shanghai Enzyme-linked Biotechnology Co., Ltd	Cat#: ml059835
Chicken IL-4 ELISA Kit	Shanghai Enzyme-linked Biotechnology Co., Ltd	Cat#: ml059838
Chicken IL-6 ELISA Kit	Shanghai Enzyme-linked Biotechnology Co., Ltd	Cat#: ml059839
Chicken IL-10 ELISA Kit	Shanghai Enzyme-linked Biotechnology Co., Ltd	Cat#: ml059830
BCA Protein Assay Kit	Solarbio	Cat#: PC0020
E.Z.N.A.® Soil DNA Kit (for cecal digesta)	Omega Bio-Tek	Cat#: D5625-01

**Deposited data**		

16S rRNA sequencing data	This paper	PRJNA1233905

**Experimental models: Organisms/strains**		

Yellow-feathered broilers (Gallus gallus domesticus), male	Chuang Yu Poultry Breeding Co.	N/A

**Oligonucleotides**		

Primers for RT-qPCR (see Table 2 for sequences)	Sangon Biotech (Shanghai, China)	This paper

**Software and algorithms**		

SPSS 26.0	IBM	https://www.ibm.com/products/spss-statistics
ImageJ	N/A	https://imagej.nih.gov/ij/
Origin 2022	OriginLab	https://www.originlab.com/
QIIME2 (for 16S data analysis)	N/A	https://qiime2.org/

### Experimental model and study participant details

#### Animal ethics statement

Experimental protocols and treatments were approved by the Institutional Animal Care and Use Committee of Shihezi University (A2023-245).

#### Broilers

A total of 480 1-day-old male, yellow-feathered broilers were randomly divided into four groups with six replicates and 20 broilers per replicate. On days 1–21 of the experiment (EFR stage), broilers were allowed *ad libitum* access to feed for 24 h (control group, group C), 20 h (group F), 16 h (group E), and 12 h (group T) per day. Broilers in all groups were fed *ad libitum* for 24 h per day on days 22–70 of the experiment (ADF stage). The experiment lasted for 70 days. All broilers were housed in individually ventilated cages under specific pathogen–free (SPF) conditions, with a 12 h light/dark cycle and *ad libitum* access to water. Each cage initially housed 20 broilers, which was reduced to 19 after sampling on day 21. All groups of broilers were fed the basal diet purchased from the Xinjiang Tiankang Feed Co., Ltd. (Xinjiang, China), which was formulated based on the Chinese agricultural industry standard “Nutritional requirements for yellow-feathered broiler chicks” (NY/T 3645–2020). Diet composition and nutrient contents are listed in Table 4.

**Table 4:** Ingredient composition and nutrient contents of basal diets

Items	Content		
	1–21 days	22–42 days	43–70 days
**Ingredients, %DM**			

Corn	60.08	58.98	56.54
Soybean meal	9.50	–	–
Cottonseed meal	10.00	10.00	7.00
Wheat middling	–	5.00	10.00
Barley grain	5.00	5.00	5.00
Corn gluten meal	5.50	3.50	3.00
Corn distillers dried grains with solubles	3.00	3.00	3.00
Corn gluten meal	–	4.50	4.50
Feather meal, hydrolyzed	1.00	1.50	2.00
Meat and bone meal	–	3.00	3.00
Lard	1.00	2.00	3.00
Premix^a^	4.92	3.52	2.96
Total	100.00	100.00	100.00

**Nutrient levels,**^b^**%DM**			

ME/(MJ/kg)	12.35	12.77	13.19
CP	19.50	17.50	16.50
Ca	0.90	0.80	0.70
TP	0.58	0.49	0.47
Lys	1.08	0.98	0.88
Met+Cys	0.76	0.71	0.65

a The premix provided the following per kg of diets: 1 to 21 days of age, CaHPO4 11.5 g, Lys 8.4 g, Met 2.1 g, Thr 1.5 g, Trp 0.3 g, Ile 0.2 g, Val 0.2 g, VA 12,000 IU, VD3 600 IU, VE 45 IU, VK 2.5 mg, VB1 2.4 mg, VB2 5.0 mg, VB6 2.8 mg, VB12 16 μg, nicotinic acid 42 mg, pantothenic acid 12 mg, folic acid 1.0 mg, biotin 0.12 mg, choline 1,300 mg, Cu 7 mg, Fe 80 mg, Zn 80 mg, I 0.7 mg, Mn 60 mg, Se 0.15 mg; 22 to 42 days of age, Lys 8.9 g, Met 2.1 g, Thr 1.5 g, Trp 0.4 g, Ile 0.4 g, Val 0.4 g, VA 9,000 IU, VD3 500 IU, VE 35 IU, VK 2.2 mg, VB1 2.3 mg, VB2 5.0 mg, VB6 2.4 mg, VB12 15 μg, nicotinic acid 35 mg, pantothenic acid 10 mg, folic acid 0.7 mg, biotin 0.1 mg, choline 1,000 mg, Cu 7 mg, Fe 80 mg, Zn 60 mg, I 0.6 mg, Mn 60 mg, Se 0.15 mg; 43 to 70 days of age, Lys 7.6 g, Met 1.7 g, Thr 1.0 g, Trp 0.3 g, VA 9,000 IU, VD3 500 IU, VE 35 IU, VK 2.2 mg, VB1 2.3 mg, VB2 5.0 mg, VB6 2.4 mg, VB12 15 μg, nicotinic acid 35 mg, pantothenic acid 10 mg, folic acid 0.7 mg, biotin 0.1 mg, choline 1,000 mg, Cu 7 mg, Fe 80 mg, Zn 60 mg, I 0.6 mg, Mn 60 mg, Se 0.15 mg.

b CP and ME are measured values and others are calculated values.

### Method details

#### Sample collection and the calculations of growth performance and immune organ indices

Body weight and feed intake were recorded. On days 21 and 70, one bird per replicate (close to average group weight) was euthanized for sample collection. Serum, ileum, and cecal digesta were collected and stored. Immune organs (liver, thymus, spleen, bursa of Fabricius) were excised and weighed. ADG, ADFI, FCR, and immune organ indices were calculated according to the following formulas:

$$\text{ADG}\left(g/d\right)=\frac{\left[\text{final}\;\text{weight}\;\left(g\right)-\text{initial}\;\text{weight}\;\left(g\right)\right]}{\text{number}\;\text{of}\;\text{days}\;\left(d\right)}$$

$$\text{ADFI}\;\left(g/d\right)=\frac{\text{total}\;\text{feed}\;\text{intake}\;\left(g\right)}{\text{number}\;\text{of}\;\text{days}\;\left(d\right)}$$

$$\text{FCR}=\frac{\text{feed}\;\text{intake}\;\left(g\right)}{\left[\text{final}\;\text{weight}\;\left(g\right)-\text{initial}\;\text{weight}\;\left(g\right)\right]}$$

$$\text{Immune}\;\text{organ}\;\text{index}\;\left(g/\text{kg}\right)=\frac{\text{immune}\;\text{organ}\;\text{weight}\;\left(g\right)}{\text{live}\;\text{body}\;\text{weight}\;\left(\text{kg}\right)}$$

#### Serum enzyme-linked immunosorbent assay (ELISA)

Concentrations of serum TNF-α, IL-1β, IL-4, IL-6, IL-10, IgA, IgG, and IgM were determined using commercially available chicken-specific ELISA kits (see key resources table), according to the manufacturers’ instructions.

#### Hematoxylin and eosin (H&E) staining

Ileum samples were fixed in 4% paraformaldehyde. They were embedded, sectioned, and stained with H&E. Histomorphologic changes in the stained samples were observed and photographed with a 20× light microscope (BX51; Olympus, Tokyo, Japan) equipped with a digital camera (DP72; Olympus, Tokyo, Japan). Three fields of view were selected from each ileal section, villus height (VH) and crypt depth (CD) were measured, and the ratio of villus height to crypt depth (VH/CD) was calculated.

#### 16S rRNA gene sequencing and analysis

Total genomic DNA was extracted from cecal digesta using a commercial DNA extraction kit (see key resources table). The V3-V4 region of the bacterial 16S rRNA gene was amplified with primers 338F and 806R. Libraries were sequenced on an Illumina HiSeq 2500 platform. Sequence data were processed and analyzed using QIIME2. Alpha diversity (Chao1, Shannon) and beta diversity (Bray-Curtis distance, visualized by PCoA) were assessed. Differentially abundant taxa were identified using Linear Discriminant Analysis Effect Size (LEfSe) with an LDA score >2.0.

#### Cecal SCFA analysis

Cecal content samples were thawed at room temperature, and a portion (0.30 ± 0.05 g) was homogenized in 1500 μL of ultrapure water by vortexing for 30 s. The homogenate was centrifuged at 5,000 × g for 4 min at 25 °C. Subsequently, 500 μL of the supernatant was transferred to a new tube, deproteinized with 100 μL of 25% metaphosphoric acid solution, vortexed for 30 s, and centrifuged at 15,000 × g for 15 min at 25 °C. The final upper layer was transferred into a vial and utilized for the determination of SCFAs via gas chromatography (Agilent 7890 B, USA).

#### Quantitative reverse transcription polymerase chain reaction analysis (RT-qPCR)

Total RNA was extracted from ileum tissue, reverse transcribed to cDNA, and analyzed by qPCR using gene-specific primers (sequences in Table 5). Gene expression levels were normalized to β-actin and calculated using the 2^−ΔΔCt^ method.

**Table 5:** Primer sequences used in RT-qPCR

Gene	Forward sequence (5′–3′)	Reverse sequence (5′–3′)	Gene bank no.
*TNF-α*	TTGGAAGCAGCGTTCGGGAGT	TTGTGGGACAGGGTAGGG	NM_204267.2
*IL-1β*	GAGATGGCGTTCGTTCCC	CACGAAGCACTTCTGGTTGA	NM_204524.2
*IL-4*	AACATGCGTCAGCTCCTGAAT	TCTGCTAGGAACTTCTCCATTGAA	NM_001398460.1
*IL-6*	CGCCTTTCAGACCTACCT	ACCACTTCATCGGGATTT	NM_204628.2
*IL10*	GGTAGTTTTCAGTCTTTGGTTG	CCAGGTCTTGGGATGTTC	NM_001004414.4
*Wnt3*	GACCATTCTGGACCACATG	CTCGGAAGCACTGTCGTATT	NM_001081696.2
*Lrp5*	GCATGGCACTTTTACTGGCATC	TCATATAAGGGGGCACCACTGC	NM_001012897.2
*Lgr5*	TGACGGGGCTACATGG	TCCACTGGAAAGACCCA	XM_046909876.1
*β-catenin*	GGAACGAGACAGCGGATCT	CCGCCAGAGTGGAAAG	NM_205081.3
*β-actin*	ATTGTCCACCGCAAATGCTTC	AAATAAAGCCATGCCAATCTCGTC	NM_205518.2

#### Western blotting (WB)

Ileal proteins were extracted using RIPA lysis buffer. Protein concentration was determined by BCA assay. Equal amounts of protein were separated by SDS-PAGE and transferred to PVDF membranes. Membranes were blocked and incubated with primary antibodies (see key resources table for details and dilutions) overnight at 4°C, followed by incubation with fluorescent secondary antibodies. Protein bands were visualized using an Odyssey infrared imaging system and quantified with ImageJ software. β-actin was used as a loading control.

### Quantification and statistical analysis

Measurement data are expressed as means ± standard error of mean (SEM). A two-way analysis of variance (ANOVA) was performed to assess the main effects of feeding regimen (C, F, E, T), time (21 days vs. 70 days), and their interaction. For data at each time point, if a significant main effect was found, Tukey’s honest significant difference (HSD) post hoc test was used for multiple comparisons among groups (one-way ANOVA analysis was used to calculate body weight on day 1, with the Tukey’s HSD applied). *p* < 0.05 was considered statistically significant. All these analyses were conducted using SPSS 26.0 software.

For microbiota data, Linear discriminant analysis effect size (LEfSe) was used to identify differentially abundant taxa between groups, with an LDA score threshold of >2.0. Spearman’s rank correlation analysis was performed to evaluate the relationships between the relative abundance of gut microbiota and host phenotypes. Spearman’s correlation analysis was conducted using Origin 2022 software.
